# Notes

**Published:** 1901-02

**Authors:** 


Secretary.

### NOTES.

*Beef Measles.* A disease formerly unknown to the colonies has been discovered by Veterinary Surgeon Desmond. A piece of diseased beef was sent to him for examination, and on close inspection he found a number of small cysts in the connective tissue. He examined these with the microscope, and was surprised to find that the disease was beef measles. This is the first case that has been reported in Australia, and its discovery in cattle here is recognized to be of serious importance. The subject deserves the most thorough investigation, and this it will receive at the hands of the health authorities.—*South Australian Register*, November 3, 1900.

Cape Colony, South Africa, has adopted rigid regulations relative to tuberculosis applying to the introduction of dairy or breeding cattle. A certificate of freedom from this disease is required or the animals are quarantined and tested with tuberculin by the colonial veterinarians prior to admission.

Reports reach us of a new process for branding stock in New Zealand which is said to be proving satisfactory in that colony. It consists in the application on a wooden brand of a caustic which obliterates the hair without injuring the skin. It is stated that the cost of branding one hundred cattle amounts only to fifteen shillings, which is fully compensated for by the sound skins put on the market. As cattle must be marked in some legible and indelible way, any method which may supersede the hot branding-iron will prove of great advantage.--*The Agricultural Journal*, December 6, 1900.

Veterinary Surgeon Desmond, of Adelaide, South Australia, reports an outbreak of tuberculosis among a number of pigs. The lesions were largely abdominal, and bacteriological examinations confirmed the nature of the deposits found in the liver and intestinal glands. The fact that the pigs were fed considerable offal and the location of the lesions indicated the development of the disease by ingesta.

The *Breeders' Gazette* makes a very poor defence to the very just criticism of the use of its editorial columns against the veterinary profession by Dr. S. G. Hendren, of Indianapolis, Ind. The JOURNAL has answered editorially the same attack upon the profession in its November number.

Governor Roosevelt, of New York, has recommended that the control of bovine tuberculosis and glanders be placed under the Department of Agriculture and not kept any longer under the State Board of Health.

Germany is reported to have purchased more than $1,500,000 worth of horses in California during the latter part of 1900, which were shipped to China. This was a great blessing to the Pacific Coast breeders, with their restricted home market.

Rabies was an active theme of discussion at the Keystone Veterinary Medical Association in January. The diagnosis by physical symptoms and period of incubation, and the errors in diagnosis made by veterinarians proved interesting and instructive.

Dr. J. O. George, of Camden, N. J., adds another successful result in the use of carbolic acid in tetanus in the horse.

South Carolina, in connection with its Agricultural Experiment Station, is to have an agricultural journal. Dr. G. E. Nesom, veterinarian to the station, will assist in its editorial direction.

"The State's Duty in the Prevention of Tuberculosis," by Dr. C. O. Probst, secretary of the State Board of Health of Ohio, was an interesting and important presentation of the subject before the Convention of the Representatives of Boards of Health.

Cornstalk disease was the cause of serious losses to the cattle-owners of Nebraska during the fall months.

Green sorghum poisoning of cattle is the subject-matter of *Bulletin* No. 63 of the Nebraska Experiment Station.

